# Supplementary material for: Combining Sampling Methods with Attractor Dynamics in Spiking Models of Head-Direction Systems
Source: bioRxiv. 2025 Feb 26:2025.02.25.640158. Preprint. [Version 1] doi: 10.1101/2025.02.25.640158 (PMC11888369; doi:10.1101/2025.02.25.640158)
Supplement: 1 [file NIHPP2025.02.25.640158V1-supplement-1.pdf]

bioRxiv preprint doi: https://doi.org/10.1101/2025.02.25.640158; this version posted February 26, 2025. The copyright holder for this preprint (which was not certified by peer review) is the author/funder, who has granted bioRxiv a license to display the preprint in perpetuity. It is made available under aCC-BY 4.0 International license.

631 # Supplementary Figures

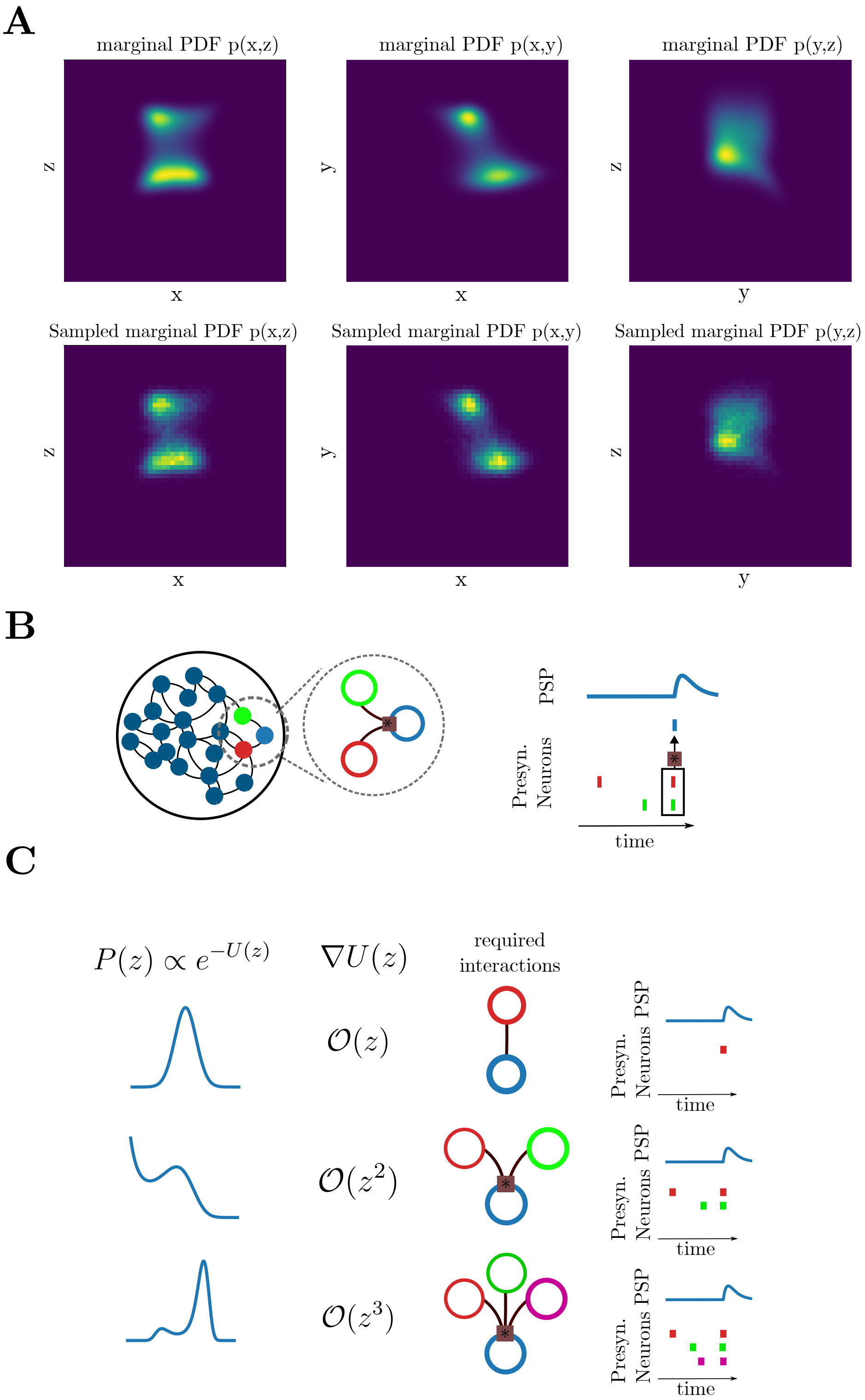

Supp. Fig. 1. Sampling from high−D non-Gaussian distributions in mSCNs
(A) $3D$, 4th order polynomial moment exponential family distribution, given by $\exp(-U(x,y,z))$, where $U(x,y,z) = x + x^2 - 3x^3 + y^2 - 3y + x^2y^2 + xyz^2 + x^2yz + x^4 + z^4$. Top row: true marginal PDF, bottom: sampled marginal normalized histogram after 10000 samples. **(B)** Nonlinear polynomial dynamical systems in SCNs require multiplicative interactions among pairs of incoming inputs. The PSP of the receiving neuron i will be affected according to $\mathbf{\Omega}_{ijk} r_t^j r_t^k$, where j, k are the two presynaptic neurons, and $\mathbf{\Omega}$ is the matrix of nonlinear synaptic weights. **(C)** Higher-order polynomials require increasingly complicated multiplicative interactions (Supp. Fig. 1B). Specifically, networks have to implement $(g-1)$-th order multiplications for distributions with g-th order polynomial moments (see Methods).

bioRxiv preprint doi: https://doi.org/10.1101/2025.02.25.640158; this version posted February 26, 2025. The copyright holder for this preprint (which was not certified by peer review) is the author/funder, who has granted bioRxiv a license to display the preprint in perpetuity. It is made available under aCC-BY 4.0 International license.

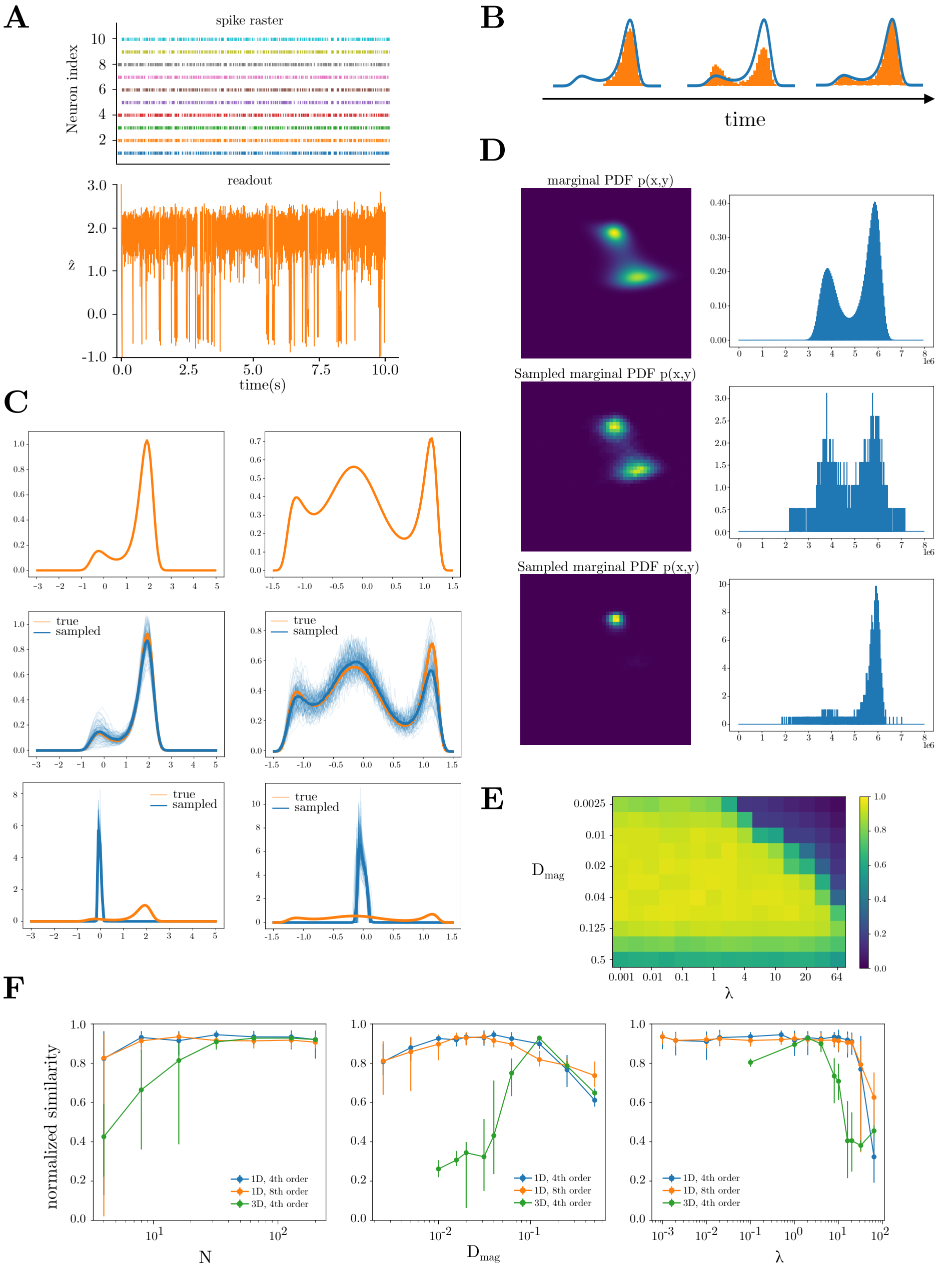

Supp. Fig. 2. Sampling behaviour and performance for various distributions
(A) Example neural activity of 10 neurons (top) and linearly read out samples (bottom) for a bimodal distribution (as in panel C, left). **(B)** Schematic of the evolution over time of an empirical histogram-based density from samples (orange), and the true density (blue). **(C)** Visualization of good and bad sampling for the 1D, 4th and 8th order distributions (left and right, respectively); true distributions (top) are sampled with best (middle) and worst (bottom) parameters as per Table 2. Orange lines are the true distributions, thick blue line is the mean of 100 runs, each plotted as a thin blue line. **(D)** Visualization of good and bad sampling for the 3D, 4th order distribution; true distribution (top) is sampled with the best and worst parameters, shown in middle and bottom respectively, as per Table 2. The left column showcases the marginal distributions $P(x, y)$, and the right column contains the flattened 3D histogram. **(E)** Similarity scores as a function of D_{mag} and λ for the 1D, 4th order distribution. **(F)** Similarity scores for characterizing the sampling performance for three distributions given by Eq. (96) with varying parameter sets. The optimal parameter set $\{N, D_{mag}, \lambda\}$ is determined for each distribution (Table 2), and consequently one parameter is varied while the other two are fixed. Blue, orange, and green line correspond to the 1D fourth order, 1D eighth order, and 3D fourth order distributions respectively. Each datapoint is the mean over 20 runs for that parameter set, and the corresponding bars span 5th to 95th percentiles. Similarity score is determined as per Perfomance calculation.

bioRxiv preprint doi: https://doi.org/10.1101/2025.02.25.640158; this version posted February 26, 2025. The copyright holder for this preprint (which was not certified by peer review) is the author/funder, who has granted bioRxiv a license to display the preprint in perpetuity. It is made available under aCC-BY 4.0 International license.

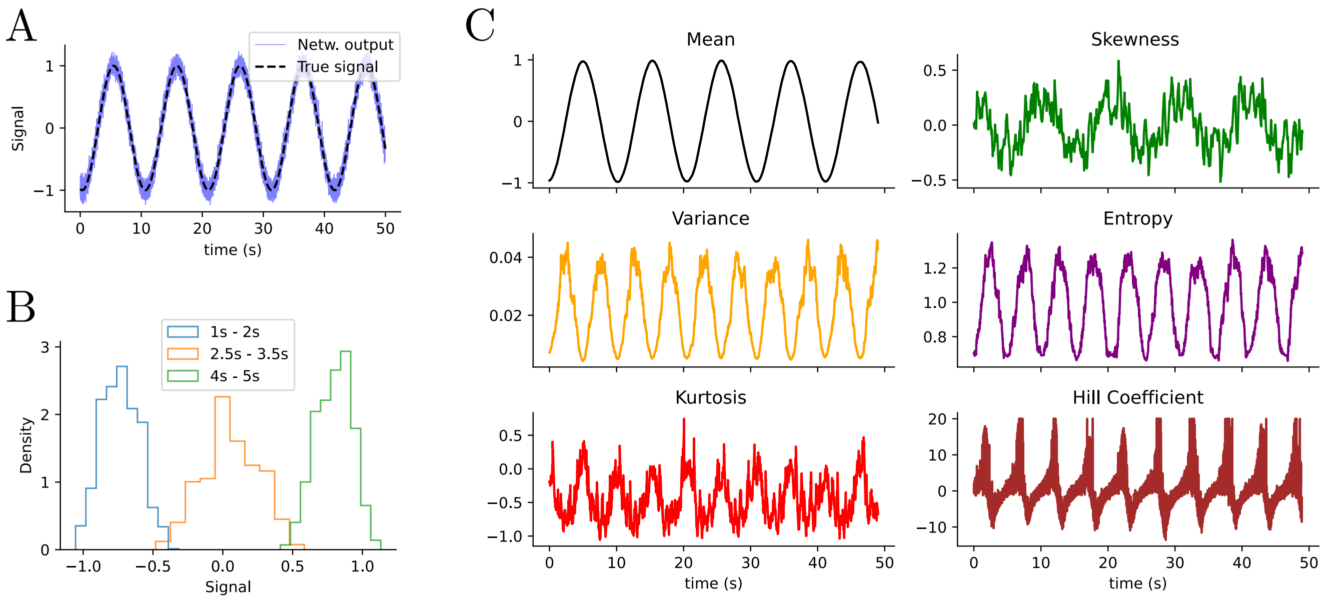

Supp. Fig. 3. Sampling-based inference allows the computation of various statistics online on time-varying stimuli posterior distributions

An oscillating external stimulus is encoded by 100 Poisson neurons for 50 seconds with a $dt = 10^{-3}$s. A SCN network with 50 neurons, following the dynamics of Eq. (53), computes real-time sampling-based inference. **(A)** True signal (dashed black) vs network output (light blue) over 50 seconds. **(B)** We assume that a downstream reader computes various statistics on a running window of 1000 samples. Three histograms show the real-time sampling process. **(C)** Various statistics measured on the running windows of 1000 samples: first 4 central moments, entropy measured on the discretized distribution, hill coefficient measured with a hill estimator with $k = 100$.

bioRxiv preprint doi: https://doi.org/10.1101/2025.02.25.640158; this version posted February 26, 2025. The copyright holder for this preprint (which was not certified by peer review) is the author/funder, who has granted bioRxiv a license to display the preprint in perpetuity. It is made available under aCC-BY 4.0 International license.

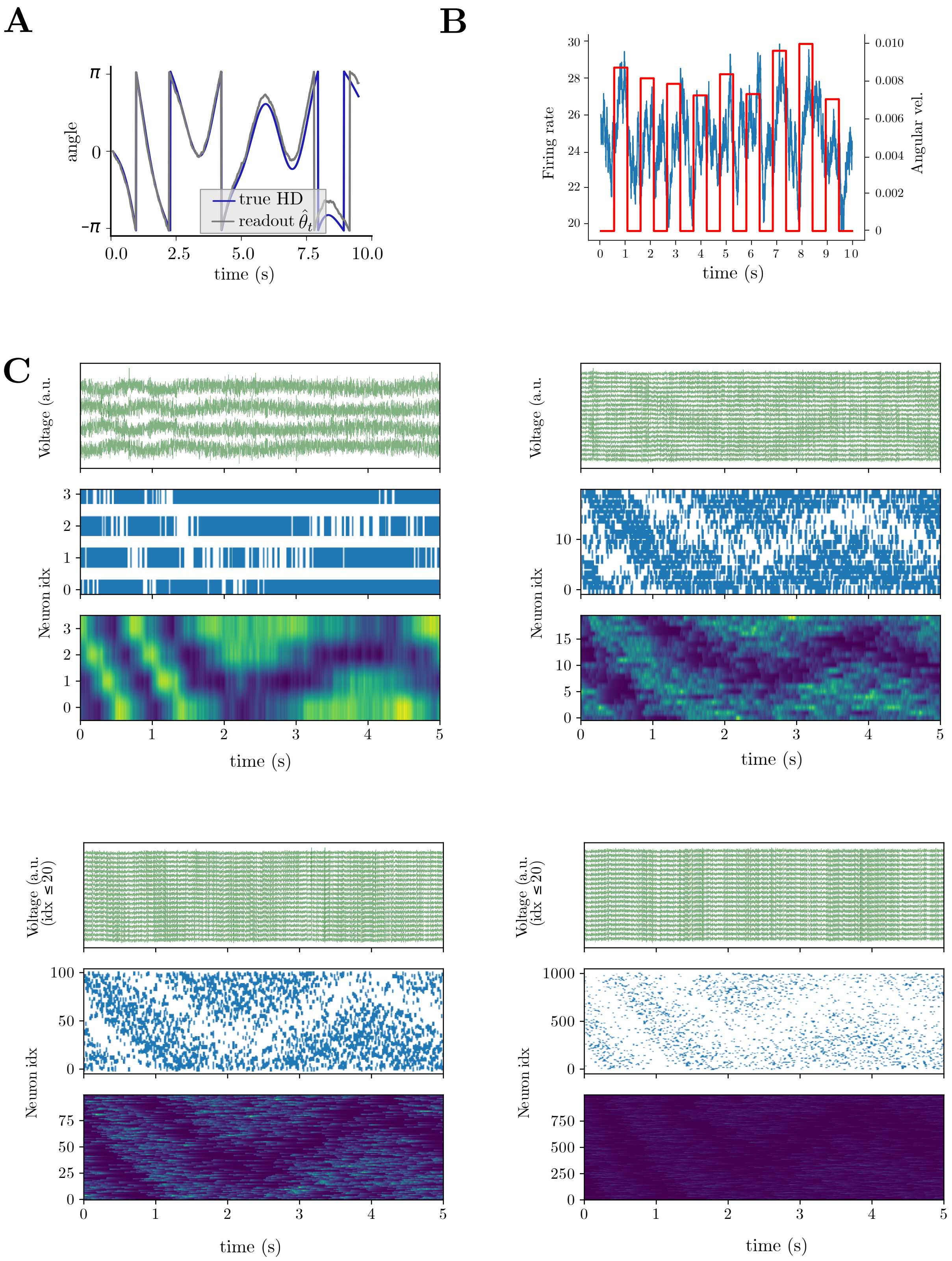

Supp. Fig. 4. Biological phenomena captured by sampling-based HD network
(A) True HD vs SCN readout for an example network run as in Fig. 4. Notice that the network accumulates errors and loses the true orientation over time. **(B)** Average network activity (blue) during pulses of angular velocity (red). **(C)** Voltage (**v**), spikes (o), and filtered spikes (r) for SCN HD networks with 4, 16, 100, and 1000 neurons. Same inputs as in Fig. 4.

bioRxiv preprint doi: https://doi.org/10.1101/2025.02.25.640158; this version posted February 26, 2025. The copyright holder for this preprint (which was not certified by peer review) is the author/funder, who has granted bioRxiv a license to display the preprint in perpetuity. It is made available under aCC-BY 4.0 International license.

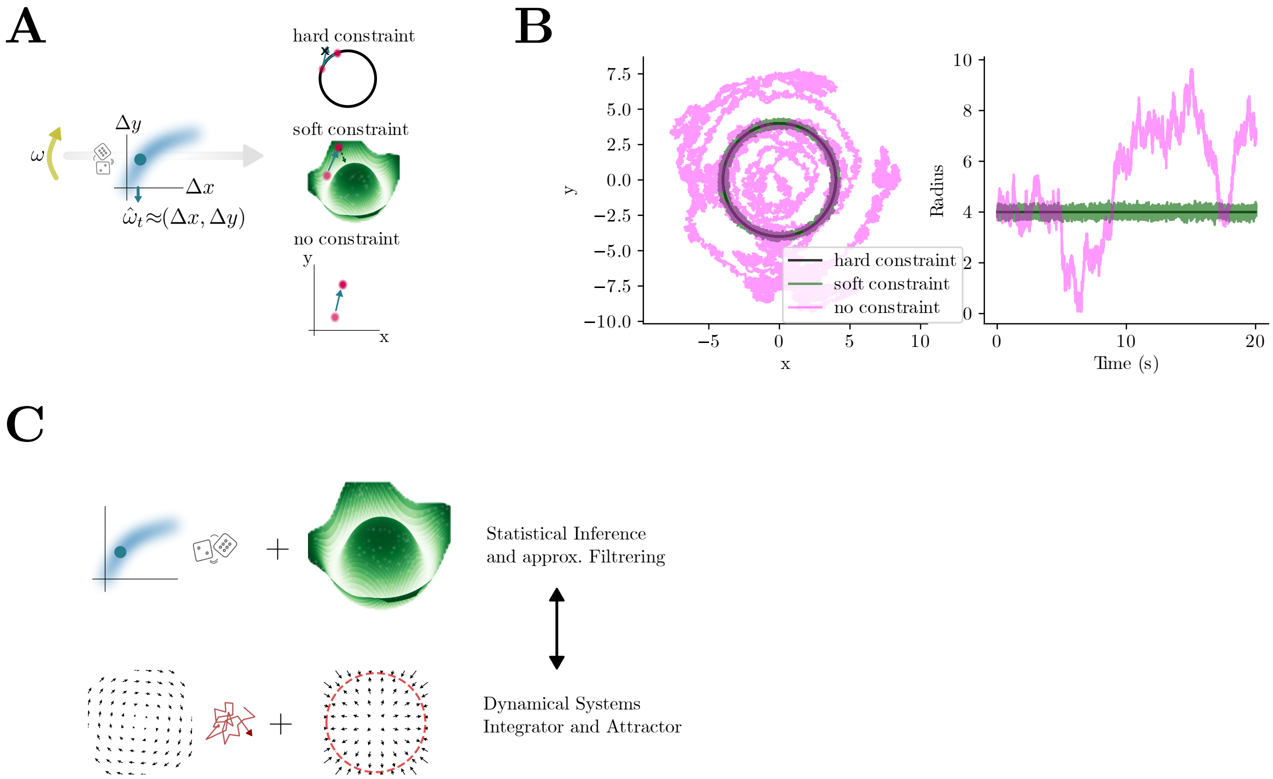

Supp. Fig. 5. Constraining $2-D$ angular representations.
(A) Left: angular velocity ω is encoded noisily, and then transformed into a $2-D$, $\Delta x, \Delta y$ HD update. Right: the HD representation in $2-D$ can be constrained in various ways; top: a hard constrain represents a renormalization, whereby $x_t, y_t = \rho\frac{x_t}{\sqrt{x_t^2+y_t^2}}, \rho\frac{y_t}{\sqrt{x_t^2+y_t^2}}$; middle: a soft constraint biases the dynamics to return towards the circle by including an energy term away from the circle, as in $(x^2+y^2)(x^2+y^2-\rho^2)$, so that the dynamics are driven towards the outside if $x^2+y^2<\rho^2$, and towards the inside if $x^2+y^2>\rho^2$; bottom: no constraints allow the dynamics to go wherever in the $2-D$ plane. **(B)** Example x, y dynamics for hard (red), soft (green), and no constraints (cyan). Notice the radius oscillations (right panel). **(C)** Writing the approximate Baysian filtering problem through Langevin dynamics results into a nonlinear dynamical system resembling a $2-D$ integrator combined with a circular limit cycle (i.e. attractor).

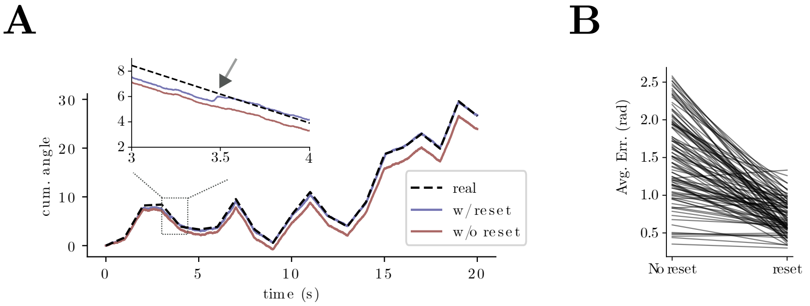

Supp. Fig. 6. Implementing a visual reset mechanism through multimodal integration
(A) Comparison of cumulative angle for real vs. HD with reset vs. HD without reset over 20 seconds behavior. Inset: zoom in to show the effect of the reset. **(B)** Avg. error after 20 seconds behavior with vs. without reset mechanisms.
